# Hierarchically Labeled Database Indexing Allows Scalable Characterization of Microbiomes

**DOI:** 10.1016/j.isci.2020.100988

**Published:** 2020-03-17

**Authors:** Filippo Utro, Niina Haiminen, Enrico Siragusa, Laura-Jayne Gardiner, Ed Seabolt, Ritesh Krishna, James H. Kaufman, Laxmi Parida

**Affiliations:** 1 IBM Research, T.J. Watson Research Center, Yorktown Heights, NY 10598, USA; 2 IBM Research, The Hartree Centre, Warrington, WA4 4AD, UK; 3 IBM Research, Almaden Research Center, San Jose, CA 95120, USA

**Keywords:** Microbiology, Microbial Genetics, Bioinformatics

## Abstract

Increasingly available microbial reference data allow interpreting the composition and function of previously uncharacterized microbial communities in detail, via high-throughput sequencing analysis. However, efficient methods for read classification are required when the best database matches for short sequence reads are often shared among multiple reference sequences. Here, we take advantage of the fact that microbial sequences can be annotated relative to established tree structures, and we develop a highly scalable read classifier, PRROMenade, by enhancing the generalized Burrows-Wheeler transform with a labeling step to directly assign reads to the corresponding lowest taxonomic unit in an annotation tree. PRROMenade solves the multi-matching problem while allowing fast variable-size sequence classification for phylogenetic or functional annotation. Our simulations with 5% added differences from reference indicated only 1.5% error rate for PRROMenade functional classification. On metatranscriptomic data PRROMenade highlighted biologically relevant functional pathways related to diet-induced changes in the human gut microbiome.

## Highlights

• Microbiome function can be characterized with respect to an annotation hierarchy
• An efficient method was developed for functional classification of sequencing reads
• Direct lowest taxonomic unit assignment enabled improved classification time
• Biologically relevant pathways were revealed from metatranscriptomic sequencing data

## Graphical Abstract

**Figure:**
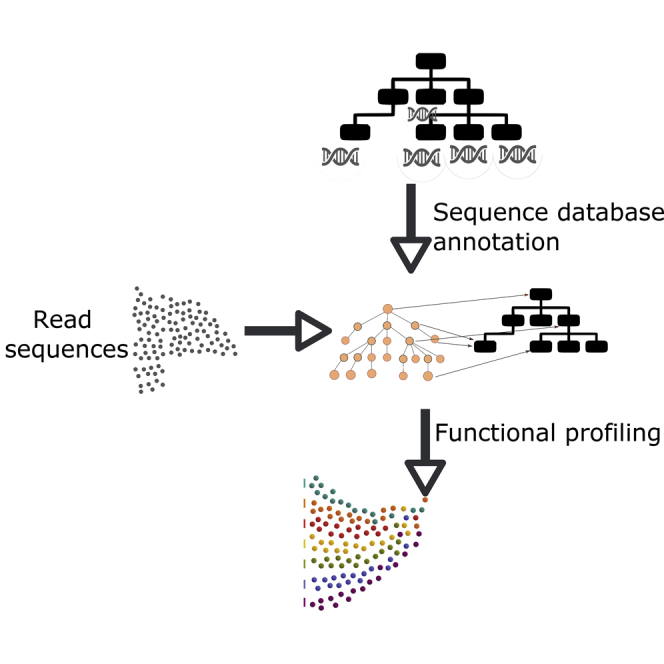

## Introduction

Microbiome analysis involves determining the composition and function of the community of microorganisms in a particular locale (Claesson et al., 2017). Variation in the human microbiome has been linked to numerous health conditions and diseases such as obesity, inflammatory bowel disease, cancer, and neurodegenerative diseases (Elinav et al., 2019, Gilbert et al., 2018). In addition to identifying the members of a diverse microbial community, an important task is understanding the biological processes that can occur in that community. Determining the metabolic processes performed by microbes, and their related host interactions, is critical for understanding how the microbiome functions, and eventually for perturbing disease-related processes. Functional annotation has been approached, for example, by alignment-based approaches matching metagenomic and metatranscriptomic sequencing reads against functionally annotated protein databases (Franzosa et al., 2018, Zhu et al., 2018) and other methods as discussed by, e.g., Knight et al. (2018) and Niu et al. (2017).

Microbial reference databases typically contain many sequences that are distinct yet highly similar, resulting in reads frequently matching multiple sequences equally well. Furthermore, microbial sequences can be organized in terms of a hierarchical annotation tree, e.g., a taxonomy of genomes or functional units. In light of these observations, an optimal strategy would thus assign reads directly to the relevant *lowest taxonomic unit* (LTU) in a taxonomic tree. This paper focuses on efficient functional classification of microbiome sequencing reads in terms of a *functional taxonomy* (such as KEGG enzyme codes Kanehisa et al., 2017). The challenge is to efficiently and accurately assign reads to the relevant LTU, given a large database of sequences that have been annotated with a functional taxonomy.

Rapid methods for classifying microbiome reads against a phylogenetic taxonomy (e.g., NCBI reference taxonomy Pruitt et al., 2007) have been introduced, such as Kraken (Wood and Salzberg, 2014) and Kraken 2 (Wood et al., 2019) that employ a *k*-mer index, i.e., a hash-based full-text index for patterns of *fixed* length *k*. This has the drawback of operating on fixed-length substrings of the read and then reconciling their matches to assign the read in the context of the taxonomy. The value of *k* also needs to be determined prior to building the search index, and *k*-mer reduction may be required with large databases to reduce the search index size. Kaiju (Menzel et al., 2016), on the other hand, allows searching for variable-length exact matches in amino acid databases, with speed and accuracy comparable with or better than with methods using fixed size *k*-mers. However, answering an LTU query with Kaiju involves post-processing, and the time required depends on the number of alternative matches.

We propose a highly scalable method, PRROMenade, that combines efficient variable-length sequence classification with direct assignment of a read to its LTU node in the annotation hierarchy. PRROMenade thus utilizes the desirable aspects of both Kaiju and Kraken. To accomplish scalable classification, we employ the *generalized Burrows-Wheeler transform* (GBWT) (Burrows and Wheeler, 1994). We present a method to annotate generalized suffix arrays (Manber and Myers, 1990) and bidirectional BWTs (Schnattinger et al., 2012) in order to answer LTU queries in constant time for patterns of *arbitrary* length (see Transparent Methods and Figure S1).

We applied PRROMenade with the OMXWare database (Seabolt et al., 2019) and the KEGG enzyme function taxonomy (Kanehisa et al., 2017). In addition to simulated data confirming PRROMenade accuracy, we applied it on experimental metatranscriptome sequencing data to demonstrate its applicability in functional characterization of human microbiomes. As many as 67% of examined metatranscriptomic reads matched multiple database sequences equally well, indicating that direct hierarchical labeling could improve classification performance. Indeed, PRROMenade took less than half the time of Kaiju to classify experimental reads.

PRROMenade supports analyzing high-throughput metagenomic and metatranscriptomic sequencing experiments, in conjunction with large-scale reference databases (nucleotide or amino acid) and hierarchies for naming or functional annotation. Furthermore, our annotation method is not limited to microbiome annotation and can be used for general-purpose string annotation.

## Results

### Database Indexing

Building the PRROMenade index on the OMXWare database (3.7 billion AA from 11.9 million protein domains) took approximately 11 h (on single core; indexing approximately 3 h and annotation approximately 8 h) and peak memory of 60 GB. The finished index had size 57 GB. On the GS database (1.1 million AA from 2,810 proteins), index building took approximately 3 min and peak memory of 70 MB. Timing could be further improved by employing parallelization techniques.

### Accuracy on Simulated Data

PRROMenade classified all simulated reads on the OMXWare database. On the reads containing 5% sequencing errors, classification speed was 9.4 million reads/min, PRROMenade assigned 91.5% to the correct functional node (87.8%) or its non-root ancestor (3.7%), with 7.1% assigned to the root and only 1.5% incorrectly assigned reads. The average MEM length was 17.0 AA. In order to test situations where sequences are highly divergent from the reference database, we also simulated reads with 10% and 30% errors (differences from database sequences) to represent divergent organisms. For these data, 11.0% and 32.7% of read assignments were erroneous, respectively, indicating a linear relationship between sequence divergence and classification error rate. The average MEM length decreased to 11.4 AA and 8.4 AA, respectively. For the metatranscriptomic reads average MEM was 8.3 AA, resembling the reads with 30% errors.

### Hierarchical Database Structure

The premise of this work is that protein domain sequences are more similar within subtrees of the functional hierarchy than between subtrees. One way to demonstrate this is to examine the assignments for perfect simulated reads. Since almost all database sequences are placed at leaf nodes in the tree, if reads are assigned to internal nodes it indicates shared sequences across the subtree below that node. The distribution of simulated read assignments compared with database sequences (Figure S2) shows enrichment for assignments of perfect reads particularly immediately above the leaf level (level 3; 3.9% reads versus 1.0% database content). This indicates shared sequence content in the corresponding subtrees, supporting the premise of hierarchical functional classification. The fraction of reads assigned to the root increases with the read error rate, and the metatranscriptomic data distribution resembles most that of reads with 30% errors (Figure S2).

### Redundancy in the Database

We examined the degree of redundancy in the OMXWare protein domain database in terms of multiple MEMs during read classification. On simulated data with 5% sequencing errors, 10% of the reads would need an LTU computation, with an average 14.6 alternative MEMs per read. On the experimental metatranscriptomic data, 67% of the reads would need an LTU computation, with on average 11.7 alternative MEMs per read. PRROMenade avoids the LTU computation in these cases, on one hand by assigning each MEM to its corresponding node in the hierarchy directly (instead of all the individual sequences where it appears) and on the other hand by selecting a random representative in case of multiple equally long MEMs. Multi-matching is becoming an even greater issue in practice, with the number of related sequences in reference databases increasing. For example, there are currently over 200,000 assembled *Salmonella* strains in EnteroBase (Alikhan et al., 2018).

### Comparison with Alignment-Based Approach

Alignment-based approach mi-faser (Zhu et al., 2018) with their GS database was compared with PRROMenade using the same database. On the simulated 50k read pairs (with 5% errors), mi-faser was orders of magnitude slower, taking over 20 min (0.00236 million reads/min), whereas PRROMenade took less than 1 s (11.2 million reads/min). In terms of accuracy, mi-faser classified 98.9% of the reads, with 98.9% of the answers to the correct function, 0.9% to an ancestor and 0.2% root (when considering the multi-mapping reads), with 0.001% errors. PRROMenade classified all reads, with 98.3% assignments to the correct function, 0.2% to an ancestor, and 1.1% to the root, with only 0.4% errors (mean MEM of 16.3 AA). However, on the experimental metatranscriptomic data mi-faser classified fewer than 1% of the reads, which combined with the classification speed makes mi-faser unusable in practice for this type of data.

### Application on Metatranscriptomic Data

Classification speed on metatranscriptome reads from a study of diet-related changes in the fecal microbiome David et al. (2014) was 15.4 million reads/min for PRROMenade, compared with 6.3 million reads/min for Kaiju. PRROMenade assigned 38% of reads to a functional category and 62% to the root.

**Figure 1:**
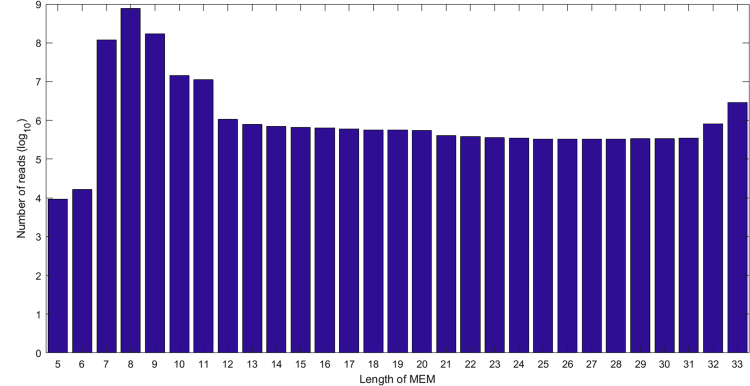
Distribution of Maximal Exact Match Lengths Distribution of maximal exact match lengths on experimental metatranscriptomic data (5–33 AA) against the OMXWare database. Average MEM length is 8.3 AA.

The average size of the maximal exact matches on OMXWare database was 8.3 AA (25 nt), see Figure 1, shorter than a typical *k*-mer indexing length of 31 nt. In fact, the fraction of reads with a match of 10 AA or longer was only 2.3%. Increased flexibility is indeed needed when matching reads to microbial databases inevitably missing many sequences detected in sampled microbiomes.

**Figure 2:**
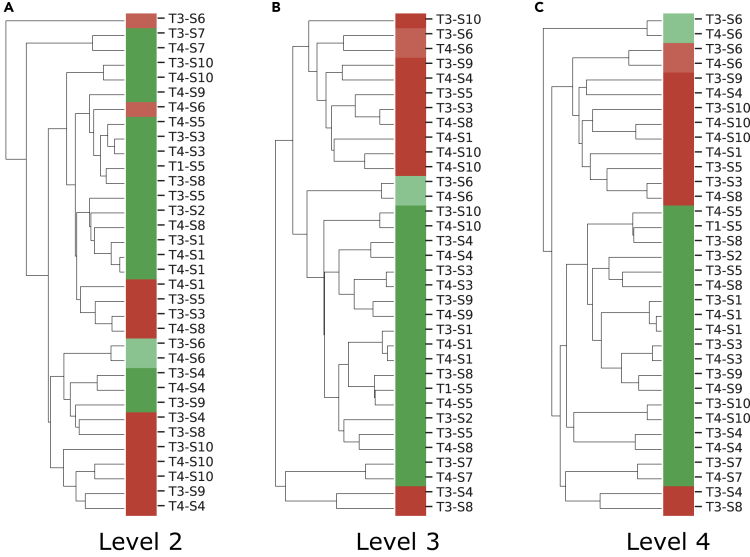
Clustering of Functional Profiles Clustering of metatranscriptome PRROMenade profiles for plant- (green) and animal-based (red) diet samples at various levels of functional hierarchy. The vegetarian subject (S6) samples are denoted with a lighter shade.

Clustering of the functional profiles shows better agreement with diet labels at levels 3 and 4 compared with level 2 (Figure 2). The leaf-level (level 4) clustering provides a clear separation between the diets, with only two animal-based diet samples (T3-S4 and T3-S8: time T3 subjects S4 and S8) intermixed with the plant-based cluster (also observed in the original publication). Animal-based diet sample T3-S5 clusters here with other animal-based diet samples, contrary to the original publication. Samples from the same subject tend to cluster together, in particular the replicate samples for T4-S1 and T4-S10. The vegetarian subject's (S6) plant-based samples outlie the main clusters, indicating different functionality compared with all other samples.

**Figure 3:**
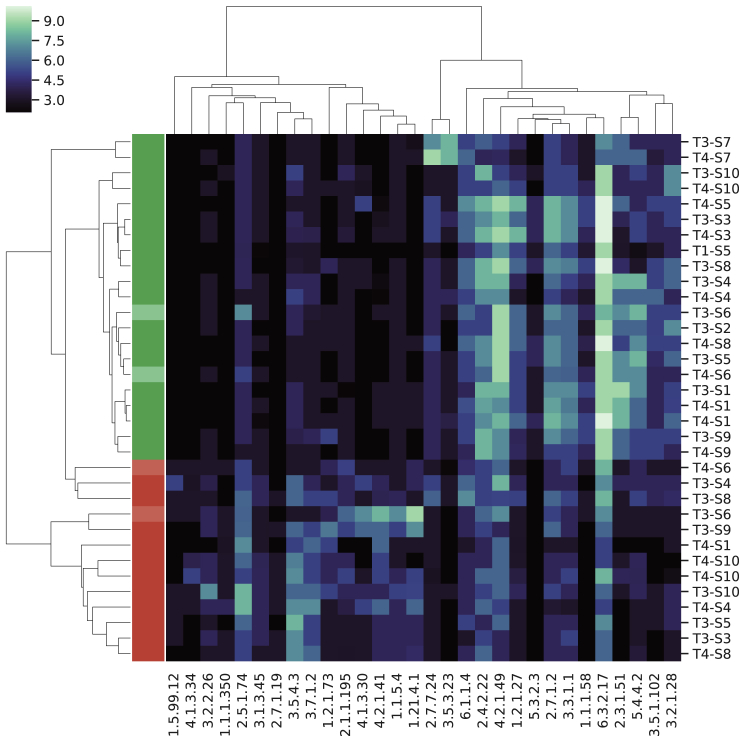
Clustering of Differentiating Functions Clustering of top 30 differentially abundant functions at level 4 (columns) and of samples (rows), colored by RoDEO projected values; the left cluster shows functions enriched in animal-based diet and right cluster those enriched in plant-based diet.

The top differential functions are visualized in Figure 3. Although there are commonalities among the associated differential metabolic pathways and the original analysis of the data (David et al., 2014), we discovered additional functional changes in the microbiome during plant- and animal-based diets. Many of the discovered pathways relate to amino acid catabolism and biosynthesis and therefore overlap those previously noted for these data. Our analysis indicated enrichment in the plant-based diet for biosynthesis of secondary metabolites, biosynthesis of antibiotics, arginine and proline metabolism, and starch and sucrose metabolism. Streptomycin biosynthesis was an additional discovery from our analysis compared with the previous study. Streptomycin (an antibiotic) is produced by *Streptomyces* bacteria that are abundant in soils and enriched in the root microbiomes of many different plant species (Newitt et al., 2019). In the animal-based diet we detected enrichment for propanoate metabolism and microbial metabolism in diverse environments. Additionally, we detected enrichment for purine metabolism, which fits as animal-based foods tend to have higher purine content (Jakše et al., 2019).

## Discussion

We propose high-throughput sequencing read classification with respect to a functional hierarchy. To accomplish efficient classification, we propose a labeled indexing approach, implemented as PRROMenade, that directly provides the lowest taxonomic unit (LTU) of the maximal exact match (MEM) for a query sequence. The LTU assignment is achieved in constant time for a query sequence of any length, once the MEM has been located. This makes read classification more efficient as it avoids locating all possible multiple matches for a query and instead reports their LTU directly. PRROMenade improves on the state of the art for the problem of sequence labeling, especially when faced with databases riddled with multiple near-identical sequences such as closely related microbial strains.

Our simulated experiments demonstrate that read classification in the context of a functional hierarchy with PRROMenade is efficient and accurate. Although in simulations read classification error rate scaled with the sequence divergence rate, on experimental data we detected meaningful and previously unreported differences between functional profiles of metatranscriptomes obtained for plant- and animal-based diet groups. On the experimental data the majority of sequence reads had multiple database matches and thus benefitted from the direct labeling approach, making PRROMenade more than twice as fast as Kaiju.

With very large datasets required to capture the natural microbial sequence diversity, there is a need for scalable approaches like PRROMenade. Functional classification enables advancing beyond naming of organisms to providing insights into the functional capacity of a microbiome based on high-throughput sequencing experiments. As future work, parallelizing the search index construction would allow more rapid updates of the reference, which may be required as databases keep increasing in size.

The proposed annotation method can be used in conjunction with nucleotide sequence databases in addition to protein databases and phylogenetic hierarchies in addition to functional hierarchies. Furthermore, the approach is not confined to applications on biological taxonomies and can be used for general-purpose sequence annotation.

### Limitations of the Study

In this work we chose to assign the reads based on their maximal exact match to the database, using a relatively short minimum length threshold. The threshold could be increased to reduce the number of false positives, as the simulated experiments indicated error rate scales with sequence reads' divergence from the database. However, we observed that the resulting functional assignments still separated samples based on their phenotype and provided insights into the functional differences in the respective microbial communities.

PRROMenade was designed as a rapid method for labeling query sequences in terms of an annotation hierarchy. It can separate sequences into those that do not match the database, those that are assigned to the leaf and internal nodes in the hierarchy, and those that are assigned to the root and are thus not informative. For the reads in the last category, a post-processing step could be used with a slower, more sensitive method such as alignment to possibly yield additional assignments.

## Methods

All methods can be found in the accompanying Transparent Methods supplemental file.

## Data and Code Availability

All datasets used in this work are available from publicly available sources as cited in the manuscript. PRROMenade is implemented in C++ using the SeqAn library (Reinert et al., 2017). The executable is available at https://github.com/ComputationalGenomics/PRROMenade.

## Supplemental Information

Document S1. Transparent Methods and Figures S1 and S2

## References

[bib1] Alikhan N.F., Zhou Z., Sergeant M.J., Achtman M. (2018). A genomic overview of the population structure of *Salmonella*. PLoS Genet..

[bib2] Burrows, M., Wheeler, D.J., 1994. A block-sorting lossless data compression algorithm. Technical Report 124. Digital SRC Research Report.

[bib3] Claesson M.J., Clooney A.G., O’Toole P.W. (2017). A clinician’s guide to microbiome analysis. Nat. Rev. Gastroenterol. Hepatol..

[bib4] David L.A., Maurice C.F., Carmody R.N., Gootenberg D.B., Button J.E., Wolfe B.E., Ling A.V., Devlin A.S., Varma Y., Fischbach M.A. (2014). Diet rapidly and reproducibly alters the human gut microbiome. Nature.

[bib5] Elinav E., Garrett W., Trinchieri G., Wargo J. (2019). The cancer microbiome. Nat. Rev. Cancer.

[bib6] Franzosa E.A., McIver L.J., Rahnavard G., Thompson L.R., Schirmer M., Weingart G., Lipson K.S., Knight R., Caporaso J.G., Segata N., Huttenhower C. (2018). Species-level functional profiling of metagenomes and metatranscriptomes. Nat. Methods.

[bib7] Gilbert J.A., Blaser M.J., Caporaso J.G., Jansson J.K., Lynch S.V., Knight R. (2018). Current understanding of the human microbiome. Nat. Med..

[bib8] Jakše B., Jakše B., Pajek M., Pajek J. (2019). Uric acid and plant-based nutrition. Nutrients.

[bib9] Kanehisa M., Furumichi M., Tanabe M., Sato Y., Morishima K. (2017). KEGG: new perspectives on genomes, pathways, diseases and drugs. Nucleic Acids Res..

[bib10] Knight R., Vrbanac A., Taylor B., Aksenov, Callewaert C., Debelius J., Gonzalez A., Kosciolek T., McCall L.I., McDonald D. (2018). Best practices for analysing microbiomes. Nat. Rev. Microbiol..

[bib11] Manber, U., Myers, G., 1990. Suffix arrays: a new method for on-line string searches, in: SODA ’90: Proceedings of the First Annual ACM-SIAM Symposium on Discrete Algorithms, pp. 319–327.

[bib12] Menzel P., Ng K.L., Krogh A. (2016). Fast and sensitive taxonomic classification for metagenomics with Kaiju. Nat. Commun..

[bib13] Newitt J.T., Prudence S.M.M., Hutchings M.I., Worsley S.F. (2019). Biocontrol of cereal crop diseases using *Streptomycetes*. Pathogens.

[bib14] Niu S.Y., Yang J., McDermaid A., Zhao J., Kang Y., Ma Q. (2017). Bioinformatics tools for quantitative and functional metagenome and metatranscriptome data analysis in microbes. Brief. Bioinform..

[bib15] Pruitt K.D., Tatusova T., Maglott D.R. (2007). NCBI reference sequences (RefSeq): a curated non-redundant sequence database of genomes, transcripts and proteins. Nucleic Acids Res..

[bib16] Reinert K., Dadi T.H., Ehrhardt M., Hauswedell H., Mehringer S., Rahn R., Kim J., Pockrandt C., Winkler J., Siragusa E. (2017). The SeqAn C++ template library for efficient sequence analysis: a resource for programmers. J. Biotechnol..

[bib17] Schnattinger T., Ohlebusch E., Gog S. (2012). Bidirectional search in a string with wavelet trees and bidirectional matching statistics. Inf. Comput..

[bib18] Seabolt E., Nayar G., Krishnareddy H., Agarwal A., Beck K., Terrizzano I., Kandogan E., Roth M., Mukherjee V., Kaufman J. (2019). OMXWare, a cloud-based platform for studying microbial life at scale. arXiv.

[bib19] Wood D., Lu J., Langmead B. (2019). Improved metagenomic analysis with kraken 2. Genome Biol..

[bib20] Wood D.E., Salzberg S.L. (2014). Kraken: ultrafast metagenomic sequence classification using exact alignments. Genome Biol..

[bib21] Zhu C., Miller M., Marpaka S., Vaysberg P., Ruhlemann M.C., Wu G., Heinsen F.A., Tempel M., Zhao L., Lieb W. (2018). Functional sequencing read annotation for high precision microbiome analysis. Nucleic Acids Res..

