# Supplementary material for: Hierarchically Labeled Database Indexing Allows Scalable Characterization of Microbiomes
Source: iScience. 2020 Mar 17;23(4):100988. doi: 10.1016/j.isci.2020.100988 (PMC7125348; doi:10.1016/j.isci.2020.100988)
Supplement: Document S1. Transparent Methods and Figures S1 and S2 [file mmc1.pdf]

iScience, Volume *23*

Supplemental Information

Hierarchically Labeled Database Indexing Allows Scalable Characterization of Microbiomes

Filippo Utro, Niina Haiminen, Enrico Siragusa, Laura-Jayne Gardiner, Ed Seabolt, Ritesh Krishna, James H. Kaufman, and Laxmi Parida

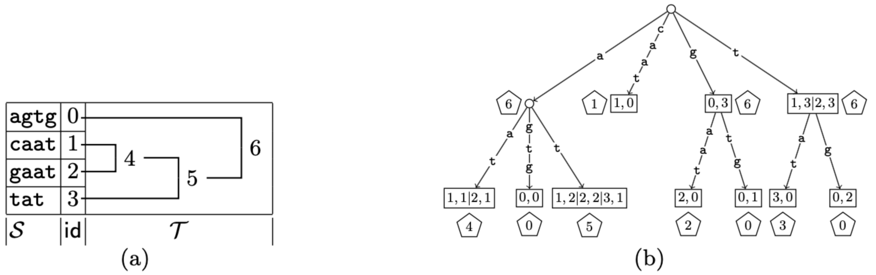

		gsa		
i	tax	id	pos	$\mathcal{S}_{gsa}$
0	–	1	1	aat
1	4	2	1	aat
2	–	0	0	agtg
3	5	1	2	at
4	–	2	2	at
5	6	3	1	at
6	–	1	0	caat
7	6	0	3	g
8	–	2	0	gaat
9	–	0	1	gtg
10	6	1	3	t
11	–	2	3	t
12	–	3	2	t
13	–	3	0	tat
14	–	0	2	tg

(c)

			GBWT($\mathcal{S}$)		GBWT($\overline{\mathcal{S}}$)	
i	tax	id	gbwt	$\mathcal{S}_{gsa}$	gbwt	$\overline{\mathcal{S}}_{gsa}$
0	–	–	g	$\$_0$	a	$\$_0$
1	–	–	t	$\$_1$	c	$\$_1$
2	–	–	t	$\$_2$	g	$\$_2$
3	–	–	t	$\$_3$	t	$\$_3$
4	–	–	c	aat$\$_1$	g	a$\$_0$
5	4	–	g	aat$\$_2$	t	aac$\$_1$
6	–	0	$\$_0$	agtg$\$_0$	t	aag$\$_2$
7	5	–	a	at$\$_1$	a	ac$\$_1$
8	–	–	a	at$\$_2$	a	ag$\$_2$
9	6	–	t	at$\$_3$	t	at$\$_3$
10	–	1	$\$_1$	caat$\$_1$	a	c$\$_1$
11	6	–	t	g$\$_0$	a	g$\$_2$
12	–	2	$\$_2$	gaat$\$_2$	t	ga$\$_0$
13	–	0	a	gtg$\$_0$	$\$_0$	gtga$\$_0$
14	6	–	a	t$\$_1$	a	t$\$_3$
15	–	–	a	t$\$_2$	$\$_1$	taac$\$_1$
16	–	–	a	t$\$_3$	$\$_2$	taag$\$_2$
17	–	3	$\$_3$	tat$\$_3$	$\$_3$	tat$\$_3$
18	–	0	g	tg$\$_0$	g	tga$\$_0$

(d)

Figure S1: **Illustration of a taxonomic database and LTU assignment**, related to Figure 1. (a) Example of taxonomic database $(\mathcal{S}, \mathcal{T})$. The LTU between 1 and 3 is 5; the LTU between 0 and 4 is 6; (b) Generalized suffix tree of $\mathcal{S}$ annotated for LTU queries on $\mathcal{T}$. Non-terminal nodes are round while terminal nodes are squares. LTU annotations are shown inside pentagons. The LTU of pattern a is 6 while the LTU of aa is 4. (c) Generalized suffix array of $\mathcal{S}$ annotated for LTU queries on $\mathcal{T}$. Pattern agtg corresponds to a singleton with interval $[2, 3)$ and its LTU is $\mathsf{id}[2] = 0$. Pattern a corresponds to a leftmost node with interval $[0, 6)$ and its LTU is $\mathsf{tax}[5] = 6$. Pattern at is not on a singleton nor on a leftmost node, its interval is $[3, 6)$ and its LTU is $\mathsf{tax}[3] = 5$; and, (d) Generalized bidirectional BWT of $\mathcal{S}$ annotated for LTU queries on $\mathcal{T}$. Note that here we are showing the terminating character \$ for illustrative purposes, while in practice we are not using it.

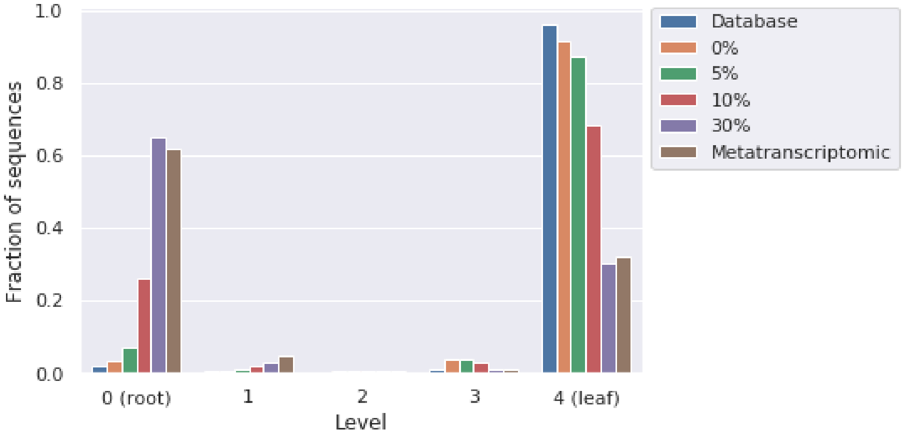

Figure S2: **Visualization of the functional hierarchy level assignments**, related to Figure 1. Distribution of OMXWare database sequence content, and of PRROMenade assignments of simulated reads with 0% to 30% errors representing divergence from the refrence, on the functional hierarchy tree from root to leaf level. In addition, metatranscriptomic read assignments are shown.

Transparent Methods

Lowest taxonomic unit problem

In the following, we describe how sequence database indexing with associated taxonomic labeling is performed using a generalized Burrows-Wheeler transform (GBWT) (Burrows and Wheeler, 1994). Here *taxonomy* refers to a microbial naming hierarchy or a functional hierarchy, organized as a tree. The database may contain either nucleotide or amino acid sequences, in the method description below we show an example using the four-letter nucleotide alphabet, for convenience.

Consider a taxonomic database $(\mathcal{S}, \mathcal{T})$ consisting of a collection of strings $\mathcal{S} = \{s_0, s_1, \ldots, s_{m-1}\}$ of total length n over the ordered alphabet $\Sigma = \{\mathtt{a}, \mathtt{c}, \mathtt{g}, \mathtt{t}\}$ and a taxonomic tree $\mathcal{T}$, i.e., a rooted tree with m leaves labeled by the indexes of strings in $\mathcal{S}$ and internal nodes referring to taxonomic units. Given a pattern string p over Σ, the problem is to retrieve the *lowest taxonomic unit* (LTU) in $\mathcal{T}$ where p occurs. Figure S1(a)shows an illustration. We denote lowest taxonomic

unit (LTU) similarly to the definition of lowest common ancestor of nodes u and v in $\mathcal{T}$ by $\textsc{lca}_{\mathcal{T}}(u, v)$ and the iterated LCA as:

$$\textsc{lca}_{\mathcal{T}}(u, \ldots, y, z) = \textsc{lca}_{\mathcal{T}}(u, \textsc{lca}_{\mathcal{T}}(\ldots, \textsc{lca}_{\mathcal{T}}(y, z))). \tag{1}$$

We define $\textsc{ids}(v) = \{k : (k, l) \in \textsc{suf}(v)\}$. The LTU of node v with $\textsc{cld}(v) = \{w_0, w_1, \ldots, w_{k-1}\}$ is:

$$\textsc{ltu}_{\mathcal{T}}(v) = \textsc{lca}_{\mathcal{T}}(\textsc{ids}(v), \textsc{ltu}_{\mathcal{T}}(w_0), \textsc{ltu}_{\mathcal{T}}(w_1), \ldots, \textsc{ltu}_{\mathcal{T}}(w_{k-1})). \tag{2}$$

We conceptualize taxonomic annotation on generalized suffix trees (Gusfield, 1997) and then show how to annotate generalized suffix arrays and bidirectional BWTs.

Generalized suffix tree

We first explain our LTU annotation algorithm on a conceptual level using generalized suffix trees. Description of practical implementations using generalized suffix arrays and generalized Burrows-Wheeler transforms follow. We adopt a practical definition of generalized suffix tree (GST) that is based on terminal nodes in addition to leaves and branching internal nodes, opposed to what traditionally done in (Gusfield, 1997). Our definition is analogous to that in (Cazaux et al., 2014) and allows us to work on arbitrary collections of strings without introducing sentinel characters, e.g., `$`. In what follows, we denote the l-th suffix of string s_k as $\mathcal{S}_{(k,l)} := s_k[l] \ldots s_k[|s_k| - 1]$ where $|s_k|$ is the length of string s_k.

The generalized suffix tree of $\mathcal{S}$, abbreviated as GST($\mathcal{S}$), is a lexicographically ordered tree data structure having one node designated as the root. Each node v of GST($\mathcal{S}$) provides the following operations: $\textsc{cld}(v)$ returns the nodes children of v; $\textsc{suf}(v)$ returns a list of pairs air (k, l) refers to suffix $\mathcal{S}_{(k,l)}$; $\textsc{label}(v)$ returns a string over Σ or the empty string if v is the root; the representative of a node v, $\textsc{repr}(v)$ returns $\textsc{repr}(u) \cdot \textsc{label}(v)$ where u is the parent of v or the empty string if v is the root. We say that node v is a leaf

if $|\textsc{cld}(v)| = 0$ and internal otherwise; node v is terminal if $|\textsc{suf}(v)| \geq 1$ and non-terminal otherwise.

The GST($\mathcal{S}$) has the following properties: (i) for each (k, l) in $\textsc{suf}(v)$, $\textsc{repr}(v)$ spells exactly $\mathcal{S}_{(k,l)}$; (ii) each leaf is terminal; (iii) each non-terminal node v is branching, i.e., $|\textsc{cld}(v)| \geq 2$, and $\textsc{label}(w)$ for all $w \in \textsc{cld}(v)$ begin with distinct characters.

As each suffix in $\mathcal{S}$ is associated with one terminal node, GST($\mathcal{S}$) has at most n terminal nodes. Also, because of the branching property, GST($\mathcal{S}$) has at most $n-1$ non-terminal nodes. Therefore, we have to annotate at most $2n-1$ nodes.

We now describe a conceptual annotation of GST($\mathcal{S}$) in order to answer *lowest taxonomic unit* (LTU) queries in constant time given node v. Intuitively, the LTU can be defined as the lowest common ancestor Gusfield (1997) between any two units on the taxonomic tree. Before annotating GST($\mathcal{S}$), we preprocess $\mathcal{T}$ to answer LTU queries in constant time. In practice, we reduce LTU queries to *range minimum queries* (Bender and Farach-Colton, 2000). Subsequently, we compute the LTU for all nodes of GST($\mathcal{S}$) in a single post-order traversal. The annotation of GST($\mathcal{S}$) thus takes $\mathcal{O}(n)$ time. Figure S1(b) shows an example of annotated GST.

Generalized suffix array

The *generalized suffix array* (GSA) (Manber and Myers, 1990; Shi, 1996) of $\mathcal{S}$ is a pair $(\mathsf{id}, \mathsf{pos}) := \mathsf{gsa}$ of tables of length n where $\mathsf{id}[i] = k$, $\mathsf{pos}[i] = l$ and $\mathsf{gsa}[i] = (k, l)$. Table gsa represents a permutation of all pairs (k, l) referring to suffixes $\mathcal{S}_{(k,l)}$ in $\mathcal{S}$. Pairs in gsa are ordered $\mathcal{S}_{\mathsf{gsa}[i-1]} <_{lex} \mathcal{S}_{\mathsf{gsa}[i]}$ for all $i \in [1, n)$.

In fact, table gsa corresponds to the pre-order concatenation of $\textsc{suf}(v)$ for all terminal nodes v in GST($\mathcal{S}$). Each node v of GST($\mathcal{S}$) is univocally identified by an half-open interval $[\textsc{lb}(v), \textsc{rb}(v))$ on table gsa. $\textsc{lb}(v)$ is defined as the smallest index l of GSA for which $S_{gsa}[l]$ starts with $\textsc{repr}(v)$. $\textsc{rb}(v)$ is defined as the greatest index $r > l$ for which $S_{gsa}[r-1]$ starts with $\textsc{repr}(v)$. Each node v of GST($\mathcal{S}$) can be determined by binary searching $\textsc{repr}(v)$ on gsa. GST($\mathcal{S}$) corresponds to a recursive partitioning of gsa: the

root node corresponds to interval $[0, n)$; if v is an internal node with $\textsc{cld}(v) = \{w_0, w_1, \ldots, w_{k-1}\}$ then its children intervals are $[\textsc{lb}(v) + |\textsc{suf}(v)|, \textsc{rb}(w_0))$, $[\textsc{rb}(w_0), \textsc{rb}(w_1))$, …, $[\textsc{rb}(w_{k-1}), \textsc{rb}(v))$.

GSA($\mathcal{S}$) can be traversed in linear-time, bottom-up using the additional lcp table (Kasai et al., 2001) and top-down using lcp and child tables (Abouelhoda et al., 2004). If top-down traversal is bounded to relatively short patterns, binary search on table gsa is a practical alternative. GSA($\mathcal{S}$) supports pattern search in time $\mathcal{O}(|p| \log n)$ using only table gsa, $\mathcal{O}(|p| + \log n)$ using table lcp and $\mathcal{O}(|p|)$ using lcp (Manber and Myers, 1990) and child tables (Abouelhoda et al., 2004).

We now describe our method to store and retrieve LTUs in constant time once we reach a node v. Traversal on GST($\mathcal{S}$) to compute LTUs is readily translated onto GSA($\mathcal{S}$). The problem is how to store and retrieve annotations in GSA($\mathcal{S}$). We say that leaf v is singleton if $\textsc{lb}(v) = \textsc{rb}(v) - 1$. Furthermore, we say that a node v with parent u is leftmost if $\textsc{lb}(u) = \textsc{lb}(v)$; we determine this condition in constant time by remembering the parent u of v while traversing GSA($\mathcal{S}$) top-down. Note that we define the root to be non-leftmost. We introduce a table tax of size n to store the annotations of all non-singleton nodes. We annotate the LTU of node v as:

$$\textsc{ltu}_{\mathcal{T}}(v) = \begin{cases} \mathsf{id}[\textsc{lb}(v)] & \text{if } v \text{ is singleton,} \\ \mathsf{tax}[\textsc{rb}(v) - 1] & \text{if } v \text{ is leftmost,} \\ \mathsf{tax}[\textsc{lb}(v)] & \text{otherwise.} \end{cases} \quad (3)$$

Figure S1(c) shows an example of annotated GSA.

If v is singleton, its LTU is already annotated in table id at position $\textsc{lb}(v)$. We show by induction on GST($\mathcal{S}$) that each non-singleton node v is annotated at an available slot in tax. Prior to annotation both $\mathsf{tax}[\textsc{lb}(v)]$ and $\mathsf{tax}[\textsc{rb}(v) - 1]$ are available; after annotation one of these two slots remains available.

1. If v is a leaf. All slots in tax within interval $[\textsc{lb}(v), \textsc{rb}(v))$ are available and $\textsc{lb}(v) < \textsc{rb}(v) - 1$ as v is non-singleton. Hence, if v is leftmost $\mathsf{tax}[\textsc{lb}(v)]$ remains available, otherwise $\mathsf{tax}[\textsc{rb}(v) - 1]$ remains available.

2. If v is an internal node with children $\textsc{cld}(v) = \{w_0, w_1, \ldots, w_{k-1}\}$. Node w_{k-1} is not leftmost and $\mathsf{tax}[\textsc{rb}(w_{k-1}) - 1]$ is supposed to be available by induction. As $\textsc{rb}(w_{k-1}) = \textsc{rb}(v)$ then $\mathsf{tax}[\textsc{rb}(v) - 1]$ is available.

   (a) If v is non-terminal. We have $|\textsc{suf}(v)| = 0$, $\textsc{lb}(w_0) = \textsc{lb}(v)$ and w_0 is leftmost. By induction $\mathsf{tax}[\textsc{lb}(w_0)]$ that is $\mathsf{tax}[\textsc{lb}(v)]$ is supposed to be available.

   (b) If v is terminal. All slots in tax within interval $[\textsc{lb}(v), \textsc{lb}(v) + \textsc{suf}(v))$ are available and $\textsc{suf}(v) \geq 1$, so $\mathsf{tax}[\textsc{lb}(v)]$ is available.

   After annotation, if v is leftmost $\mathsf{tax}[\textsc{lb}(v)]$ remains available, otherwise $\mathsf{tax}[\textsc{rb}(v) - 1]$ remains available.

Our annotation method is more convenient than that one proposed by Abouelhoda et al. (2004) with the purpose of encoding suffix links on the enhanced suffix array. Essentially, Abouelhoda et al. (2004) annotate each non-singleton node v at position $\textsc{rb}(w_0) - 1$. However determining the interval of w_0 requires either binary search on gsa or access to lcp and child tables.

Generalized BWT

We denote by $\overline{s} = s[|s| - 1] \ldots s[1]s[0]$ the reversed string s and by $\overline{\mathcal{S}}$ the collection $\mathcal{S}$ with all strings reversed. We consider the ordered alphabet $\Sigma_{\$} = \{\$_1, \ldots, \$_m\} \cup \Sigma$ and append $\$_i$ to each string $s_i \in \mathcal{S}$. This is to insure that $\mathcal{S}$ is primitive, i.e., that no string in $\mathcal{S}$ is a power of some other string (Crochemore et al., 2005).

The *generalized Burrows-Wheeler transform* (GBWT) (Burrows and Wheeler, 1994) of $\mathcal{S}$ is a table gbwt of length n with:

$$\mathsf{gbwt}[i] = \begin{cases} s_{\mathsf{id}[i]}[\mathsf{pos}[i] - 1] & \text{if } \mathsf{pos}[i] > 0, \\ \$_{\mathsf{id}[i]} & \text{otherwise.} \end{cases} \tag{4}$$

Function $\textsc{rank}(c, i)$ counts the number of occurrences of character $c \in \Sigma_{\$}$ in the half-open interval $[0, i)$ of gbwt and $\textsc{lf}$ as:

$$\textsc{lf}(c, i) = \sum_{a<c} \textsc{rank}(a, n) + \textsc{rank}(c, i). \tag{5}$$

Similarly to GSA($\mathcal{S}$), each node v of GST($\mathcal{S}$) is univocally identified on GBWT($\mathcal{S}$) by an half-open interval $[\textsc{lb}(v), \textsc{rb}(v))$. This interval is now determined by searching $\textsc{repr}(v)$ backwards on gbwt using $\textsc{lf}$ (Ferragina and Manzini, 2000). For any two nodes u, v of GBWT($\mathcal{S}$) with $\textsc{repr}(v) = c\,\textsc{repr}(u)$ and $c \in \Sigma$, it holds:

$$\begin{aligned} \textsc{lb}(v) &= \textsc{lf}(c, \textsc{lb}(u) + m) - m, &(6)\\ \textsc{rb}(v) &= \textsc{lf}(c, \textsc{rb}(u) + m) - m. &(7)\end{aligned}$$

We adjust the boundaries by m since we have introduced m characters $\mathsf{\$}$ in $\mathcal{S}$. Function $\textsc{lf}$ is answered in $\mathcal{O}(1)$ time using $o(n\,|\Sigma|\log|\Sigma|)$ extra bits on top of gbwt Reinert et al. (2017). Pattern p is searched backwards in $\mathcal{O}(|p|)$ time.

The *bidirectional GBWT* (Schnattinger et al., 2012) consists of GBWT($\mathcal{S}$) and GBWT($\overline{\mathcal{S}}$) and allows searching simultaneously a pattern p backwards on GBWT($\mathcal{S}$) and its reverse $\overline{p}$ on GBWT($\overline{\mathcal{S}}$). Function $\textsc{lt}$ counts the number of characters lexicographically smaller than $c \in \Sigma_{\mathsf{\$}}$ in gbwt within interval $[i, j)$:

$$\textsc{lt}(c, i, j) = \sum_{a<c} \textsc{rank}(a, j) - \sum_{a<c} \textsc{rank}(a, i). \qquad (8)$$

If u is a node on GBWT($\mathcal{S}$), $\overline{u}$ is its corresponding node on GBWT($\mathcal{S}$) $\textsc{repr}$(u) $= \overline{\textsc{repr}}(\overline{u})$. Furthermore, if v is with $\textsc{repr}(v) = c\,\textsc{repr}(u)$, the interval of node $\overline{v}$ with $\textsc{repr}(\overline{v}) = \textsc{repr}(\overline{u})\,c$ is determined as:

$$\begin{aligned} \textsc{lb}(\overline{v}) &= \textsc{lb}(\overline{u}) + \textsc{lt}(c, \textsc{lb}(u) + m, \textsc{rb}(u) + m), &(9)\\ \textsc{rb}(\overline{v}) &= \textsc{lb}(\overline{v}) + \textsc{rb}(v) - \textsc{lb}(v). &(10)\end{aligned}$$

We construct an unidirectional BWT to answer LTU queries using only table gbwt of GBWT($\overline{\mathcal{S}}$) plus tables id and tax of GBWT($\mathcal{S}$). If we search pattern $\overline{p}$ backwards on GBWT($\overline{\mathcal{S}}$) and arrive on a node $\overline{v}$ with $\textsc{repr}(\overline{v}) = \overline{p}$, then node v corresponds to the node reached by searching p backwards on GBWT($\mathcal{S}$) or forward on GSA($\mathcal{S}$). Therefore we can still access the annotation at node v using Eq. 3. To fill tables id and tax, we traverse top-down GBWT($\overline{\mathcal{S}}$) backwards and annotate nodes on GBWT($\mathcal{S}$) forward. Top-down traversal is $\mathcal{O}(n^2)$ in the

worst case but feasible in practice if it is bounded to relatively short patterns. Figure S1(d) shows an example of annotated BWT. We remark that table id can be obtained as a byproduct of certain BWT construction algorithms (Egidi and Manzini, 2017) instead of slicing table gsa. Furthermore, tables id and tax can be sparsified when the annotation is bounded to relatively short patterns.

Read classification

In this study we employed the approach of searching for maximal exact matches (MEM) per read in an amino acid (AA) database, as does Kaiju (Menzel et al., 2016), though other approaches could be applied. Reads with MEMs shorter than 5 AA were considered unclassified. When using nucleotide reads, the read and its reverse complement are translated, obtaining in total six AA sequences corresponding to all possible reading frames. The longest of the six MEMs is used to classify the read, in case of ties the LTU of the (at most six) alternative MEMs is chosen. The MEM is used to classify the read to the corresponding LTU node in the annotation hierarchy, and the read count for that node is incremented by one.

Paired-end sequencing typically employs fragment sizes of 500 nucleotides (167 AA). However, the median domain length in the OMXWare functional database is only 283 AA and 25% of the domains are shorter than 167 AA. Therefore, for paired-end reads we first processed each read file separately and then combined the results by summing the counts per node (for PRROMenade as well as for our experiments with Kaiju).

Functional profiling

The functional profiles (counts per node) were post-processed with a "push down" approach as in (Huson et al., 2016) to summarize the counts at various fixed levels of the hierarchy, by also including the contribution of counts assigned at higher hierarchy levels. Pairwise Spearman distances were calculated, as suggested previously (David et al., 2014) (nodes assigned $< 0.1\%$ of total counts

in each sample were first discarded) and weighted linkage hierarchical clustering applied.

Functional profiles were analyzed with RoDEO (Haiminen et al., 2014) (P=10, I=100, R = 10^7), using a two-sample Kolmogorov-Smirnov test to identify top differentially abundant functions. Thirty functional codes with the lowest p-values were selected for average linkage clustering with correlation distance, and for pathway inference by mapping to functional pathways. Enriched pathways were defined as those overlapping at least two more of the top 30 functional codes for one vs. the other diet (e.g. ec00230, *Purine metabolism*, overlaps 0 top functional codes enriched in plant-based diet and 3 top functional codes enriched in animal-based diet).

Functional hierarchy and reference database

We used the KEGG Enzyme Nomenclature codes (EC) reference hierarchy (Kanehisa et al., 2017) as the functional annotation tree. EC numbers define a four-level hierarchy with seven top-level categories that we denote as child nodes of the root. For example, 2.4.1 is a third level code (2 = "Transferases", 2.4 = "Glycosyltransferases", 2.4.1 = "Hexosyltransferases"). We used the OMXWare database of bacterial protein domains (Seabolt et al., 2019), assembled and annotated from public repositories. A subset of 11.9 million OMXWare domains (of length $\geq$ 5 AA) had associated EC annotations, providing a collection of 3.7 billion total AA representing 1,130 EC identifiers. When a domain was annotated with multiple EC numbers (4% of domains), it was instead labeled with their LTU. Median protein domain length is 283 AA (mean 312 AA). PRROMenade could also be applied on, e.g., metagenome-assembled reference genomes (Pasolli et al., 2019) and other protein database such as UniProt (The Uniprot Consortium, 2018). Indeed we also applied PRROMenade on the smaller GS database of mi-faser (Zhu et al., 2018) with 1.1 million AA from 2,810 proteins.

Read simulation

Sequencing reads were simulated uniformly at random from sequences in the OMXWare and GS databases, respectively. The protein sequences were reverse translated to DNA sequences using EMBOSS backtranseq (Chojnacki et al., 2017) and sequencing reads were simulated using SAMtools WGSIM(v. 0.3.1-r13) (Li, 2011). We generated paired-end sequencing reads of length 125bp (fragment size 250nt as many database sequences were short, i.e., 25% were shorter than 500nt) with an error rate of 5% (-e 0.05) and a mutation rate of 0.1% (-r 0.001). For the OMXWare and GS databases we generated 95,614,845 and 50,009 read pairs, respectively. For OMXWare this corresponds to 1x coverage of the database sequences and inclusion of 89.5% of them (5.8% of the sequences were skipped due to having a length shorter than 150bp or 50AA), while for the smaller GS database the process resulted in 2x read coverage and inclusion of all but one database sequence.

Metatranscriptomic data

Metatranscriptomic sequencing data of fecal microbial communities during plant- and animal-based diets (David et al., 2014) was analyzed for functional read classification. A total of 59 samples from 11 subjects, one of them a life-long vegetarian (subject S6), were downloaded from the Gene Expression Omnibus (Edgar et al., 2002) (accession GSE46761). Trim Galore (Krueger, 2019) with options –length 50 –trim-n –max_n 10 was used to trim the 100 nt long paired reads. After trimming, 1.7M to 45.3M (mean 18.5M) reads per sample were retained. We filtered out potential human RNA content of up to 6.80% per sample (min 0.02%, mean 0.77%, median 0.20%), with bowtie2 (Langdon, 2015) mapping with local mode to their pre-built GRCh38 with 1K Genomes major SNPs index. All 59 samples (21 plant-based diet, 13 animal-based diet, 25 during diet transitions) were used for the match length and taxonomic level analysis, while only the 34 samples (21 plant-based, 13 animal-based) taken during the controlled diet periods (days 1–4) were used for downstream analysis shown in Figure 1.

Evaluation details

For simulated reads, if and only if the read was assigned somewhere on the path from its originating node to the root, it was recorded as correctly classified. Timing (elapsed wall clock) was recorded on an IBM SoftLayer cloud with 72 cores Intel® Xeon® Gold 6140 CPU @ 2.30GHz and 1.5TB of RAM running Ubuntu 16.04-64. Read classification was run with 72 threads. A Kaiju (Menzel et al., 2016) (v1.7.2) index was built on the OMXWare database with KEGG taxonomy, classification parameters were -a mem -m 5 -X -z 72. mi-faser (Zhu et al., 2018) (v1.52) was compared with PRROMenade on their GS database, using paired-end reads, 12 threads, and otherwise default settings (utilizing all available CPUs per thread). Classification speed is reported as the number of reads per experiment divided by total classification time (reads/min).

References

Abouelhoda, M.I., Kurtz, S., Ohlebusch, E., 2004. Replacing suffix trees with enhanced suffix arrays. Journal of discrete algorithms 2, 53–86.

Bender, M.A., Farach-Colton, M., 2000. The LCA problem revisited, in: LATIN, Springer. pp. 88–94.

Burrows, M., Wheeler, D.J., 1994. A block-sorting lossless data compression algorithm. Technical Report 124. Digital SRC Research Report.

Cazaux, B., Lecroq, T., Rivals, E., 2014. From Indexing Data Structures to de Bruijn Graphs, in: Combinatorial Pattern Matching. CPM 2014. Lecture Notes in Computer Science, pp. 89–99.

Chojnacki, S., Cowley, A., Lee, J., Foix, A., Lopez, R., 2017. Programmatic access to bioinformatics tools from EMBL-EBI update: 2017. Nucleic Acids Research 45, W550–W553.

Crochemore, M., Désarménien, J., Perrin, D., 2005. A note on the Burrows–Wheeler transformation. Theoretical Computer Science 332, 567–572.

David, L.A., Maurice, C.F., Carmody, R.N., Gootenberg, D.B., Button, J.E., Wolfe, B.E., Ling, A.V., Devlin, A.S., Varma, Y., Fischbach, M.A., Biddinger, S.B., Dutton, R.J., Turnbaugh, P.J., 2014. Diet rapidly and reproducibly alters the human gut microbiome. Nature 505, 559–63.

Edgar, R., Domrachev, M., Lash, A.E., 2002. Gene Expression Omnibus: NCBI gene expression and hybridization array data repository. Nucleic Acids Res 30, 207–210.

Egidi, L., Manzini, G., 2017. Lightweight bwt and lcp merging via the gap algorithm, in: International Symposium on String Processing and Information Retrieval, Springer. pp. 176–190.

Ferragina, P., Manzini, G., 2000. Opportunistic data structures with applications, in: Proceedings 41st Annual Symposium on Foundations of Computer Science, IEEE. pp. 390–398.

Gusfield, D., 1997. Algorithms on strings, trees, and sequences: Computer science and computational biology. Cambridge University Press, New York, NY, USA.

Haiminen, N., Klaas, M., Zhou, Z., Utro, F., Cormican, P., Didion, T., Jensen, C., Mason, C.E., Barth, S., Parida, L., 2014. Comparative exomics of Phalaris cultivars under salt stress. BMC Genomics 15 Suppl 6, S18.

Huson, D.H., Beier, S., Flade, I., Gorska, A., El-Hadidi, M., Mitra, S., Ruscheweyh, H.J., Tappu, R., 2016. MEGAN Community Edition – Interactive Exploration and Analysis of Large-Scale Microbiome Sequencing Data. PLoS Comput Biol 12, e1004957.

Kanehisa, M., Furumichi, M., Tanabe, M., Sato, Y., Morishima, K., 2017. KEGG: new perspectives on genomes, pathways, diseases and drugs. Nucleic Acids Res 45, D353–D361.

Kasai, T., Lee, G., Arimura, H., Arikawa, S., Park, K., 2001. Linear-time longest-common-prefix computation in suffix arrays and its applications, in:

Combinatorial Pattern Matching. CPM 2001. Lecture Notes in Computer Science, p. 181–192.

Krueger, F., 2019. TrimGalore. `https://github.com/FelixKrueger/TrimGalore`.

Langdon, W.B., 2015. Performance of genetic programming optimised Bowtie2 on genome comparison and analytic testing (GCAT) benchmarks. BioData Min 8.

Li, H., 2011. wgsim. `http://github.com/lh3/wgsim`.

Manber, U., Myers, G., 1990. Suffix arrays: a new method for on-line string searches, in: SODA '90: Proceedings of the first annual ACM-SIAM symposium on Discrete algorithms, pp. 319–327.

Menzel, P., Ng, K.L., Krogh, A., 2016. Fast and sensitive taxonomic classification for metagenomics with Kaiju. Nat Commun 7, 11257. URL: `https://www.ncbi.nlm.nih.gov/pubmed/27071849`, doi:`10.1038/ncomms11257`.

Pasolli, E., Asnicar, F., Manara, S., Zolfo, M., Karcher, N., Armanini, F., Beghini, F., Manghi, P., Tett, A., Ghensi, P., Collado, M., Rice, B., DuLong, C., Morgan, X., Golden, C., Quince, C., Huttenhower, C., Segata, N., 2019. Extensive unexplored human microbiome diversity revealed by over 150,000 genomes from metagenomes spanning age, geography, and lifestyle. Cell 176, e20.

Reinert, K., Dadi, T.H., Ehrhardt, M., Hauswedell, H., Mehringer, S., Rahn, R., Kim, J., Pockrandt, C., Winkler, J., Siragusa, E., Urgese, G., Weese, D., 2017. The SeqAn C++ template library for efficient sequence analysis: A resource for programmers. J Biotechnol 261, 157–168.

Schnattinger, T., Ohlebusch, E., Gog, S., 2012. Bidirectional search in a string with wavelet trees and bidirectional matching statistics. Information and Computation 213, 13–22.

Seabolt, E., Nayar, G., Krishnareddy, H., Agarwal, A., Beck, K., Terrizzano, I., Kandogan, E., Roth, M., Mukherjee, V., Kaufman, J., 2019. OMXWare, A Cloud-Based Platform for Studying Microbial Life at Scale. arXiv:1911.02095 .

Shi, F., 1996. Suffix arrays for multiple strings: A method for on-line multiple string searches, in: Jaffar, J., Yap, R.H. (Eds.), Concurrency and Parallelism, Programming, Networking, and Security. Springer. volume 1179, pp. 11–22.

The Uniprot Consortium, 2018. UniProt: a worldwide hub of protein knowledge. Nucleic Acids Research 47, D506–D515.

Zhu, C., Miller, M., Marpaka, S., Vaysberg, P., Ruhlemann, M.C., Wu, G., Heinsen, F.A., Tempel, M., Zhao, L., Lieb, W., Franke, A., Bromberg, Y., 2018. Functional sequencing read annotation for high precision microbiome analysis. Nucleic Acids Res 46, e23.
